# Helminth Infection Increases the Probability of Indeterminate QuantiFERON Gold in Tube Results in Pregnant Women

**DOI:** 10.1155/2014/364137

**Published:** 2014-02-19

**Authors:** Dawit Gebreegziabiher, Kassu Desta, Rawleigh Howe, Markos Abebe

**Affiliations:** ^1^Mekelle University, Mekelle, Ethiopia; ^2^Addis Ababa University, Addis Ababa, Ethiopia; ^3^Armauer Hansen Research Institute (AHRI), Addis Ababa, Ethiopia

## Abstract

*Background.* Approximately one-third of the world population is infected with *M. tuberculosis* and helminths (Kariminia et al. (2009), Walson et al. (2010)). Pregnancy and Helminth infection are known to suppress the T_H_1 response (Kariminia et al. (2009), Elias et al. (2006)) on which the QuantiFERON Gold in Tube (QFT-GIT) assay, that measures the released IFN-*γ* upon *in vitro* stimulation with mycobacterial antigens, relies on (Thomas et al. (2010)). *Objective.* To determine whether QFT-GIT indeterminate result is significantly associated with helminth infection or not. *Methods.* In this cross-sectional study, eighty-five pregnant mothers were screened for parasitic and LTBI using Kato-Katz and QFT-GIT test-respectively, *Result.* The prevalence of helminth infection in pregnant mothers was 23 (27%) of this 17 (20%) was due to *Schistosoma mansoni*. Among the total of 85 study participants 26.8% were QFT-GIT positive and 14 (17%) had indeterminate results. Three samples (21.4%) were randomly selected from the indeterminate QFT-GIT results and retested to check the reproducibility of the assay and remained indeterminate. QFT-GIT indeterminate result showed significant association with helminth infection. *Conclusion.* Helminth infections were significantly associated with indeterminate QFT-GIT results in pregnant mothers. Therefore further study is important to evaluate the possible effect of helminth infection by excluding the effect of pregnancy, as pregnancy also downregulates cellular immunity.

## 1. Introduction 

Tuberculosis (TB) is a leading global cause of morbidity and mortality [1–4]. About 5% of the *M. tuberculosis* infected individuals develop LTBI. This large pool of individuals with latent infection poses a major hurdle for global tuberculosis control efforts [5, 6]. Active TB results from either uncontrolled primary infection or reactivation of LTBI particularly in young children, pregnant mothers, and immune-compromised individuals [4, 5, 7, 8].

Early identification and treatment of LTBI is a proposed key strategy for global TB prevention and control. However, the lack of a gold standard test to detect LTBI makes accurate diagnosis difficult [9]. Given the delay and difficulties in reaching a microbiological diagnosis of TB disease and the lack of positive microbiology in LTBI, the diagnosis of TB infection is mostly reliant on the demonstration of immunological memory cells to mycobacterial antigens [7, 10]. Until whole blood assay such as Interferon-gamma release assay is developed, the only tool available to screen LTBI was the tuberculin skin test (TST) [11–13]. Nowadays, other *in vitro* T cell based assays for the diagnosis of LTBI are available. QFT-GIT assay is one of these assays which measures the released IFN-*γ* by sensitized T cells with specific M.TB antigens [9, 10, 12–15]. QFT-GIT is more specific than TST because it uses antigens which are encoded within region of difference 1 (early secreted antigenic target, 6 kDa (ESAT-6), and culture filtrate protein, 10 kDa (CFP-10)) and region of difference 11 (TB7.7) [10].

The prevalence of TB and helminth infection is geographically overlapping. Helminth infection is one of the factors that downregulates T_H_1 type immune response [16]. This T_H_1 to T_H_2 shift shows significant effect on T cell based immunological assays [9]. QFT-GIT detects response of effector T cells that have recently encountered antigens, though the presence of noninterpretable result, especially in developing countries, reduces its usefulness [16, 17]. Very few recent studies reported that helminth infections significantly associated with indeterminate QFT-GIT results [9], and this was also observed in this study.

## 2. Methods

A cross-sectional design was used to determine whether the indeterminate QFT-GIT assay is significantly associated with helminth infection or not. The study was conducted in Mekelle, Tigray Regional State, Northern Ethiopia, from October 2011 to July 2012. Consecutive samples were collected from 85 voluntary pregnant mothers at the last week of their ninth month of pregnancy from MCH Departments of Mekelle Hospital, Semen Health Center, and Ayder Referral Hospital. Based on the helminth and latent TB infections, our study participants were grouped into helminth and LTBI positive, helminth and LTBI negative, helminth positive and LTBI negative, and helminth negative and LTBI positive.

### 2.1. Parasitological Examination

Stool samples were collected in screw capped container and duplicate Kato slides were prepared within one hour of collection and examined within two days of collection. Wet mount preparation was also done to evaluate for hook worm and protozoan infection within 30 minutes. Following delivery, all mothers who were positive for any species of parasites were treated accordingly.

### 2.2. QuantiFERON-TB Gold In Tube Assay

All study participants were screened for LTBI using QFT-GIT, Cellestis Limited, Carnegie, Victoria, Australia. One milliliter of blood was collected into each of the three heparinized tubes: the nil (grey capped) negative control (containing only heparin), the mitogen (purple) positive control (containing phytohemagglutinin), and antigens (red capped) tube that contains *M. tuberculosis* specific antigens (ESAT-6, CFP-10, and TB7.7). The tubes were well mixed to ensure the appropriate contact of blood with antigens or mitogen and incubated at 37°C for 20 hours. Plasma was harvested by centrifuging at 3000 rpm for 15 minutes and stored at −80°C. Plasma was thawed and ELISA was performed according to the QFT-GIT kit protocol and the optical densities (OD) were measured using a microplate ELISA reader (Molecular Devices Corporation, USA) fitted with a 450 nm and 650 nm filter. The actual IFN-*γ* concentration in the sample was calculated based on the curve generated from the standards using QuantiFERON-TB Gold Analysis Software supplied by Cellestis Limited, Carnegie, Victoria, Australia.

The assay result was positive if the net IFN-*γ* response to the TB antigens was ≥0.35 IU/mL, regardless of the mitogen response, and negative if the net IFN-*γ* response was <0.35 IU/mL and the sufficient net mitogen response (>0.5 IU/mL). A result is said to be indeterminate if there was excessive IFN-*γ* production from the negative control (>8.0 IU/mL) or insufficient net IFN-*γ* response from mitogen (<0.5 IU/mL) plus net TB antigen response (<0.35 IU/mL).

### 2.3. Statistical Analysis

Data was entered and analyzed using the SPSS version 19. Descriptive statistics for sociodemographic and clinical characteristic was performed. Univariate logistic regression was used to explore the association between QFT-GIT indeterminate result and difference clinical data. Chi square (*χ* ^2^) test was also used to compare QFT-GIT indeterminate results with helminth positive and negative participants.

### 2.4. Ethical Consideration

Ethical approval was obtained from the Department of Medical Laboratory Sciences (MLS), College of Allied Health Sciences (CAHS), Addis Ababa University (AAU), and Armauer Hansen Research Institute (AHRI). Participants were enrolled in the study after they adequately understood the purpose of the study and signed in the written informed consent.

## 3. Result

### 3.1. Sociodemographic Characteristic

The median age of the study participants was 25 years (IQR 22, 28). Educational background of the study participants was primary school 50.6%, secondary school 28.2%, tertiary 10.6%, and illiterates 10.6%. All of the study participants were urban residents. Majorities (83.5%) of study participants were housewives and 11.8% were government employees.

### 3.2. Clinical Information

Forty-seven percent of the study participants had their first pregnancy, 29.41% second, and 23.5% third and above pregnancies. Of the total participants, only 15.29% were reported as BCG vaccinated. BCG scar was found only in 23.1% from the vaccinated participants. Only one participant had previous TB history eight years ago and five had previous contact with TB patients within the last 10 years. Only five of the study participants had close contact with TB patient 5 to 10 years ago (Table 1).

### 3.3. Parasitological Infection

The prevalence of helminth infection was 23 (27.06%) and the most prevalent helminths among the study participants were *Schistosoma mansoni *17 (20%) followed by *Ascaris lumbricoides *13 (15.3%), *Enterobius vermicularis* 8 (9.4%),* Trichuris trichiura* 7 (8.2%), and *Hymenolepis nana *5 (5.8%). The prevalence of protozoaninfection was 7 (8.33%), of this 6(7.24%) and 2(2.35%) was account to *Entamoeba histolytica/dispar* and *Giardia lamblia*, respectively. None of the study participants were found positive for *Schistosoma haematobium. *The overall intestinal parasite prevalence was 24 (28.2%). Among the helminth positive study participants, 31.64% had double or triple infection. Logistic regression was performed for age categories, occupation, level of educational background, and the number of pregnancies, and a significant association was observed only for educational level and parasitic infection. As the level of education increases from illiteracy to primary and then to secondary level, the odds of parasitic infection were decreased (OR = 0.50, CI = 0.211 to 0.992, and *P* = 0.048).

### 3.4. Latent Tuberculosis Infection

The result of QFT-GIT test indicated that LTBI was found in 26.83% while 17% had an indeterminate result. Three samples (21.4%) were randomly selected from the indeterminate QFT-GIT results and retested to check the reproducibility of the test which showed the same result. All indeterminate results were due to an insufficient IFN-*γ* response to mitogen. Fisher's exact test correlation coefficient was performed to explore relationship between QFT-GIT indeterminate result and helminth infection. QFT-GIT indeterminate result and helminth infection showed positive relationship (*r* = 0.13, *n* = 80, and *P* = 0.036). Although the majority of the BCG vaccinated participants (76.92%) were QFT-GIT negative, 15.38% and 7.69% had positive and indeterminate QFT-GIT results, respectively. Only 20% of the study participants who had BCG scar were positive for QFT-GIT. Only 6% of the total participants were reported to have contact with TB patients within 3 to 5 years, and 60% them had positive QFT-GIT and the remained 40% had indeterminate QFT-GIT results. Associations of QFT-GIT positivity with BCG vaccination, BCG scar, and contact with active TB patients were assessed by univariate logistic regression. The risk of LTBI was higher in participants who had contact with TB patients (OR = 2.03, CI = 0.31 to 13.51, and *P* = 0.046). BCG vaccination (*P* = 0.123), BCG scar (*P* = 0.52), previous TB history (*P* = 0.321), and pregnancy (0.409) showed no statistically significant association. The proportion of QFT-GIT indeterminate results were significantly higher in helminth positive, 10 (71.4%), participants than helminth negative, 4 (28.6%), with *χ* ^2^ = 6.06, *df* = 1 and *P* = 0.048, but this was not observed in protozoan infection (*P* = 0.273).

## 4. Discussion 

Helminth infections are highly prevalent in populations where *Mycobacterium tuberculosis* infection is also endemic. It has been widely conjectured that the strong mucosal T_H_2 and T regulatory cell responses elicited by these parasites can downregulate protective immune response against *Mycobacterium tuberculosis* [18].

This study was conducted to see if coinfection with helminth has impact on the use of QFT-GIT TB Gold assay in areas where both diseases are endemic. The prevalence of LTBI among pregnant women was 26.83% using QFT-GIT. Community based LTBI prevalence study in Afar region, Ethiopia, was higher (63.7%) [19] than our finding. Other studies in the same site reported that 59.3% of TB suspected participants were positive for LTBI, using QFT-GIT assay [20]. A study conducted at Addis Ababa University on male medical students also showed that 43.9% were QFT-GIT positive which is higher than our result [21]. One possible explanation for this might be the effect of helminth coinfection [16] and pregnancy, because pregnancy decreases cell mediated immunity and also gender difference of study participants [22].

QFT-GIT has been recommended as an alternative assay to TST to screen LTBI. QFT-GIT has better specificity and is not affected by BCG vaccination and other non-*M. tuberculosis* species infection because it uses *M. tuberculosis* specific antigens (ESAT-6/CFP-10 and TB7.7). Therefore, QFT-GIT is being used to screen contacts of TB patients to check whether contacts infected by MTB or not. Our data also showed that contacts had increased risk for LTBI (RR = 2.3, *P* = 0.046). The proportion of QFT-GIT positivity had no statistically significant association with previous BCG vaccination (*P* = 0.123) and BCG scar (*P* = 0.52) using univariate logistic regression. In contrast to our study, a research conducted in Afar region, Ethiopia reported that QFT-GIT positivity was higher in BCG vaccinated than non-vaccinated participants (76.0% versus 61.2%) [19]. A study conducted in Norway also indicated that TB exposure (OR = 1.59) and previous TB disease (OR = 11.60) had association with QFT-GIT positivity but not with BCG vaccination [23].

One of the potential drawbacks of QFT-GIT assay is the presence of indeterminate results which limits interpretation. Indeterminate results can happen due to different factors one of which is chronic helminth infection [9]. In our result, 17% of the study participants had indeterminate QFT-GIT. In addition, all of the study participants who had indeterminate QFT-GIT result were due to low or failure of cells to respond to mitogen (positive control). This was explained by effect of chronic helminth infection on the T_H_1 IFN-*γ* response as helminth infection and QFT-GIT indeterminate result showed significant positive association (*r* = 0.13, *P* = 0.036). A study conducted in Australia showed that 11% of study participants had QFT-GIT indeterminate result and all were due to the failure to respond to the positive control: the majority of them were helminth positive [7]. Another study conducted in Bangladesh also indicated that above 95% of the study participants who had indeterminate QFT-GIT results were due to insufficient response to mitogen and those were associated with helminth infection [9].

## 5. Conclusion

TB and helminth infections, which induce antagonizing immune response, are epidemiologically overlapping in developing countries; and thus helminth infection might be further complicating TB control and prevention programs by attenuating T_H_1 wings of the host immune system. Since QFT-GIT assay is also based on IFN-*γ* response from T_H_1 T cells in response to MTB antigens stimulation, the sensitivity of the assay is compromised by helminth infection. In this study, significant proportion of QFT-GIT results were categorized as indeterminate and also significantly associated with the helminth infection. Thus special consideration is required during interpretation of QFT-GIT indeterminate results in areas where helminth infection is common.

## Figures and Tables

**Table 1:** Association of indeterminate QFT-GIT results with clinical data of study participants (*N* = 85).

Characteristic	Freq. (%)	OR (95% CLI)
Number of pregnancies		
First	40 (47.5)	Reference
Second	25 (29.4)	0.23 (−0.03–3.09)
3rd and above	20 (23.5)	0.11 (−0.65–7.01)
BCG vaccination		
Yes	13 (15.3)	Reference
No	72 (84.7)	0.09 (−0.2–14.03)
BCG scar		
Yes	3 (23.1)	Reference
No	10 (76.9)	0.11 (−0.75–18)
TB history		
Yes	1 (1.18)	Reference
No	84 (98.82)	0.82 (−0.79–17)
TB contact**		
Yes	5 (5.88)	Reference
No	80 (84.12)	2.03 (0.31–13.51)

TB contact history** = contact in 5–10 years.
